# Racial and ethnic heterogeneity in diets of low-income adult females in the United States: results from National Health and Nutrition Examination Surveys from 2011 to 2018

**DOI:** 10.1016/j.ajcnut.2023.01.008

**Published:** 2023-03-03

**Authors:** Briana Joy K. Stephenson, Walter C. Willett

**Affiliations:** 1 Department of Biostatistics, Harvard T.H. Chan School of Public Health, Boston, MA, USA; 2 Departments of Nutrition and Epidemiology, Harvard T.H. Chan School of Public Health, Boston, MA, USA

**Keywords:** dietary patterns, low-income, robust profile clustering, NHANES, female, race, ethnicity

## Abstract

**Background:**

Poor diet is a major risk factor of cardiovascular and chronic diseases, particularly for low-income female adults. However, the pathways by which race and ethnicity plays a role in this risk factor have not been fully explored.

**Objectives:**

This observational study aimed to identify dietary consumption differences by race and ethnicity of US female adults living at or below the 130% poverty income level from 2011 to 2018.

**Methods:**

A total of 2917 adult females aged 20 to 80 years from the National Health and Nutrition Examination Survey (2011-2018) living at or below the 130% poverty income level with at least one complete 24-hour dietary recall were classified into 5 self-identified racial and ethnic subgroups (Mexican, other Hispanic, non-Hispanic [NH]-White, NH-Black, and NH-Asian). Dietary consumption patterns were defined by 28 major food groups summarized from the Food Pattern Equivalents Database and derived via a robust profile clustering model, which identifies foods that share consumption patterns across all low-income female adults and foods that differ in consumption patterns based on the racial and ethnic subgroups.

**Results:**

All food consumption patterns were identified at the local level, defined by racial and ethnic subgroups. Legumes and cured meats were the most differentiating foods identified across all racial and ethnic subgroups. Higher consumption levels of legumes were observed among Mexican–American and other Hispanic females. Higher consumption levels of cured meat were observed among NH-White and Black females. NH-Asian females had the most uniquely characterized patterns with a higher consumption of prudent foods (fruits, vegetables, and whole grains).

**Conclusions:**

Differences among the consumption behaviors of low-income female adults were found along racial and ethnic lines. Efforts to improve the nutritional health of low-income female adults should consider racial and ethnic differences in diets to appropriately focus interventions.

## Introduction

Poor diet is one of the largest contributing factors to cardiovascular and other chronic diseases [1]. The impact of cardiovascular disease (CVD)-related outcomes on female adults aged 35 to 54 y has increased in the past 20 years, although decreasing among other subgroups [[2], [3], [4], [5]]. This sex disparity has been suggested to result from the differences in diagnosis, care, and research specifically focused on female populations [2]. CVD research that ignores sex differences create misleading extrapolation and a gap in understanding female-specific risk factors. Amidst this sex gap, racial and economic disparities have also continued to persist. Reportedly, 45% of Black female adults have some type of CVD, compared with 32% of White female adults [6]. As CVD disparities widen, the pathways through which factors such as sex or race and ethnicity contribute to these differences are still not fully understood [7].

Dietary intake behaviors have been widely studied to identify the modifiable pathways for improving overall health and preventing chronic diseases. Many studies have focused on dietary quality using the standardized adherence scores [[8], [9], [10]]. Others have focused on individual foods/nutrients to examine the diet–disease relationships [11,12]. Owing to the intercorrelated structure of foods/nutrients and biological synergy, dietary patterns have been identified as a useful way to define exposure in diet–disease relationships, complementing those of specific foods or nutrients [13,14]. This also provides a simplified way to identify dietary behaviors of the subgroups.

Latent class models (LCM) are an easy and interpretable technique to identify the dietary patterns. However, this technique relies on a population composition where the single differentiating behavior present is diet. Populations that contain a mixture of demographics (e.g., culture, sex, and socioeconomic status) that may mediate differences in dietary behaviors pose challenges to the standard structure of LCM. The focus of the analysis is controlled by the identifiable behaviors present in the larger-sized demographic, whereas smaller-sized demographics are overlooked and dismissed as noise. We see this in the United States, where non-Hispanic (NH) White participants and/or those living above the 130% poverty income level define the majority of the population. Modeling dietary behaviors from studies sharing this makeup will yield mischaracterized behaviors of the general population and prevent us from better understanding the factors affecting the subgroups at the greatest risk for health outcomes (racial and ethnic minority and low income) [[15], [16], [17]]

Robust profile clustering (RPC) is an extension to the latent class model that can improve our understanding of the demographic-driven dietary behaviors [18]. RPC has previously been applied to identify the patterns of maternal diet by geography and Hispanic/Latino adult diet, by cultural background and US residency [18,19]. With a focus on low-income female adults, this study aimed to apply the RPC model to identify racial and ethnic differences among an at-risk demographic.

## Methods

### National Health and Nutrition Examination Survey

National Health and Nutrition Examination Survey (NHANES) is a publicly available population-based repeated cross-sectional survey. Approximately 9000 people were sampled annually from 15 unique counties of varying socioeconomic and racial and ethnic backgrounds. Survey sampling weights were provided to generate population-based estimates of collected measures. The survey was constructed to be representative of the United States using a complex survey sampling design. In 2011, the sampling strategy was revised to provide more reliable estimates of the subgroups of greater public health interest including racial minority subgroups and persons living at or below 130% poverty income level [[20], [21], [22]].

Our analysis focused on low-income female participants aged 20 y and older who self-identified themselves as Mexican–American, other Hispanic, NH-White, NH-Black, and NH-Asian American. Participants who identified themselves as Mixed/Other or those with missing race and ethnicity information were excluded. Low income was defined as those reporting a ratio of family income to poverty level at or below the 130%. Pregnant and lactating females were removed from the analysis. We pooled 4 survey cycles for the analysis (2011–2012, 2013–2014, 2015–2016, and 2017–2018). Sampling weights were adjusted for the pooled analysis in accordance with the NHANES survey methods and guidelines [[20], [21], [22]].

Dietary intake was measured using 2 24-hour dietary recalls for each participant, collected initially in person, and the second recall was collected through a telephonic call 3 to 10 days later. Participants with at least one complete dietary recall were included for the analysis. Participants with 2 complete recalls available were averaged over the 2 days. Recalls were collected as part of the What We Eat in the America survey component of NHANES [23]. USDA Food Pattern Equivalents Database converted foods and beverages reported in the Food and Nutrition Database for Dietary Studies into 37 food pattern components [[24], [25], [26], [27]]. Eight of the food pattern components were aggregated as the total major food groups (e.g., dairy, vegetable, and fruit) and were not included for the analysis. The consumption of legumes was calculated as both a vegetable (cup equivalents) and a protein source (ounces equivalent). To avoid redundancies, we excluded legumes as a vegetable source and included it as a protein source. A total of 28 food pattern components were included for this analysis (Supplemental Table 1). The levels of consumption for each food group were categorized into the following 4 levels: no consumption, low consumption (lower tertile of positive consumption), medium consumption (middle tertile of positive consumption), and high consumption (upper tertile of positive consumption), where tertiles were calculated based on the overall population [28,29]. Healthy Eating Index (HEI)-2015 scores were calculated to compare the diet patterns with diet quality using the HEI-2015 SAS macros made publicly available on the NCI website (https://epi.grants.cancer.gov/hei/sas-code.html).

CVD risk factors were included for exploratory purposes to examine whether differences in the CVD risk prevalence differed among the derived patterns. Risk factors were treated as binary outcomes. Adults with high cholesterol were defined as those with a total cholesterol level of >200 mg/dL, with an low density lipoprotein cholesterol >150 mg/dL, or who self-reported taking cholesterol-lowering medication. Adults with obesity were defined as those with BMI ≥ 30 kg/m^2^. Adults with hypertension were defined as those with an average reading of systolic blood pressure greater than 140 mg/dL, diastolic blood pressure greater than 90 mg/dL, or who self-reported medication the use of blood pressure-lowering medication. Adults with diabetes were defined as those with fasting glucose >126 mg/dL or who self-reported diabetes medication use. Adults who currently smoke were defined by yes responses to the question “SMQ040: Do you now smoke cigarettes?” Adults with complete diet data but with missing one or more CVD health data were still included for the analysis but not included in the descriptive reporting for that missing risk factor. Participation in federal assistance programs, such as women, infants, and children or Supplemental Nutrition Assistance Program, was defined by a yes response to “FSQ171: In the last 12 months, did {you/or anyone in your household} receive Food Stamp benefits?” or “FSQ162: In the last 12 months, did {you/or any member of your household} receive benefits from the WIC program, that is, the Women Infants and Children program?”

### RPC

RPC was introduced by Stephenson et al. [18] as a flexible extension of the LCM. The model breaks apart the assumption that all participants assigned to the same latent class (diet pattern) share the same consumption behaviors for all food items observed [18]. Alternatively, the RPC allowed the participants who share the overall consumption patterns of a subset of foods to assume a global pattern and participants who shared unique consumption patterns within a predefined subgroup of the population assumed a localized pattern.

The model assumed a probabilistic framework comprised of 3 components:

#### Global pattern assignment

Consistent with the standard latent class model, a global pattern described the latent dietary behaviors shared among the overall population. The assignment of the global pattern was determined through a probability vector that describes the probability of a person being assigned to one global pattern over another.

#### ***Local pattern assignment***

Within each subgroup exists another set of latent dietary behaviors that shared attributes specific to their subgroup, referred here as the local patterns. The assignment of the local pattern was determined through a probability vector that described the probability of a person from this subgroup being assigned to one local pattern over another.

#### Global/local indicator

Each food item used to describe each respective pattern has a probability of assuming the pattern detailed at the global level or the local level. This indicator, which is unique for each participant in the study population, was determined through a probability describing the likelihood of this food item to assume a global versus local pattern for that individual. The lower the probability, the more likely it was to assume a local pattern. The higher the probability, the more likely it was to assume a global pattern.

Each dietary pattern (global or local) was described with a probability matrix of *p* food item rows, where each row describes the probability of consumption at each of the *d* possible consumption levels. The modal pattern for each profile was identified by the consumption level category with the highest probability value in that row.

Mathematically, the model used the following notation. The self-reported levels of consumption of *p* unique food/beverage items by individual i∈(1,…,n), where n is the total number of participants in the study population, was defined as $y_{i}=\left(y_{i1},\ldots ,y_{ip}\right)$. The probability vector for the global and local pattern assignments was defined as $\pi =\left(\pi_{1},\ldots ,\pi_{K_{0}}\right)$ and $\lambda^{\left(s_{i}\right)}=\left(\lambda_{1}^{\left(s_{i}\right)},\ldots ,\lambda_{K_{s}}^{\left(s_{i}\right)}\right)$, respectively, where $s_{i}\in \left(1,\ldots ,S\right)$ indicated the subgroup index of individual i∈(1,…,n). The consumption *d-*length probability vector for food item j∈(1,…,p), given the assignment to global pattern *h* was defined as $\theta_{0j\cdot |h}=\left(\theta_{\left(0j1|h\right)},\ldots ,\theta_{\left(0jd|h\right)}\right)$*.* The consumption probability vector for food item j∈(1,…,p), given assignment to local pattern *l* within subpopulation index $s_{i}\in \left(1,\ldots ,S\right)$ was defined as $\theta_{1j\cdot |l}^{s_{i}}=\left(\theta_{1j1|l}^{s_{i}},\ldots ,\theta_{1jd|l}^{s_{i}}\right)$ The global/local indicator variable for individual i∈(1,…,n) and food item j∈(1,…,p) was defined as $G_{ij}$. Finally, the number of global and local patterns was defined as $K_{0}$ and $K_{s}$, respectively. The subject-specific likelihood of the RPC model was described as

$$f\left(y_{i}|s_{i},\pi ,\theta_{0},\theta_{1}^{\left(s_{i}\right)},\lambda^{\left(s\right)},G_{ij}\right)=\sum_{h=1}^{K_{0}}\pi_{h}\prod_{\begin{array}{c} j=1\\ \left(G_{ij}=0\right) \end{array}}^{p}\theta_{\left(0jr|h\right)}^{1\left(y_{ij}=r\right)}\prod_{\begin{array}{c} j=1\\ \left(G_{ij}=1\right) \end{array}}^{p}\sum_{l=1}^{K_{s}}\lambda_{l}^{\left(s_{i}\right)}\prod_{r=1}^{d}{{(\theta }_{1jr|l}^{s_{i}})}^{1\left(y_{ij}=r\right)}\;$$

Estimation of the parameters of this model was performed using a Bayesian approach. We fitted the model using a Gibbs sampling algorithm, with conditional posterior distributions described previously [18,19]. A previous distribution was required, under a Bayesian framework, to describe our previous belief on our expectation of how these parameters will behave. We assumed no previous knowledge of these estimates to allow the observed data to drive model estimation. The numbers of global and local patterns were unknown *a priori*. As a result, we overfitted the RPC model with 30 global and local patterns each so that when run using Markov Chain Monte Carlo, an interpretable set of nonempty global and local patterns remained [30]. Posterior computation, model diagnostics, previous sensitivities, and convergence were performed as described previously [18,19]. Data were preprocessed using SAS 9.4 [31]. RPC model was analyzed in Matlab 2022a. Survey sampling weights accounting for the NHANES study design were incorporated posthoc and were summarized using the survey package in R 4.0. All data and code to replicate the analysis have been made publicly available on the GitHub repository (https://github.com/bjks10/RPC/tree/master/NHANES).

## Results

**FIGURE 1:**
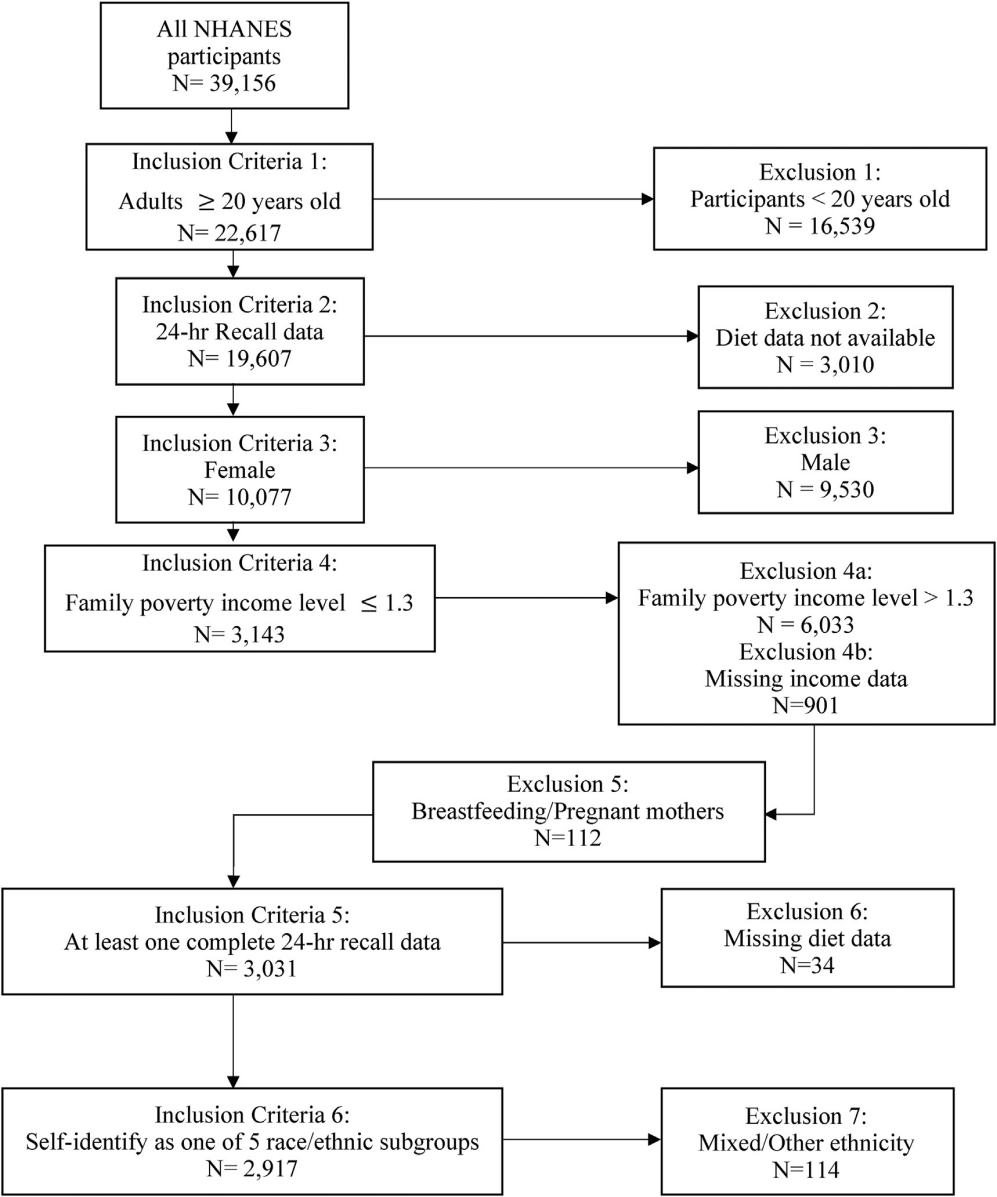
CONSORT diagram of National Health and Nutrition Examination Survey 2011 to 2018 participants included for the study analysis. A total of 2917 female adults living at or below 130% poverty income level were included after all exclusion/inclusion criteria were applied.

A total of 2917 female adult participants living at or below the 130% family poverty income level were included for the analysis of this study (Figure 1). Demographic information of these participants is provided in Table 1. Participants were mostly between the ages of 20 and 34 y (33.7%), self-identified as NH-White (49.5%), married or living with a partner (42.6%), and high school level education (43.7%). Household sizes were largest among the NH-Asian females (4.9 ± 0.8) and lowest among the NH-White females (3.3 ± 0.2). Average energy intake was highest among the NH-Black females (1828 ± 116) and lowest among the NH-Asian females (1398 ± 91). Most participants reported having high cholesterol levels. NH-Black females had the highest prevalence of high blood pressure (45.9 ± 4.0%) and obesity with a BMI ≥30 (61.7 ± 3.9%). Other Hispanic females had the highest prevalence of diabetes (19.6 ± 5.2%) and high cholesterol (58.0 ± 6.9%). NH-White females had the highest proportion of current smokers (42.0 ± 4.9%).

**TABLE 1:** Demographic information of low-income adult females pooled from 2011 to 2018

Demographic	Overall	Mexican	Other Hispanic	Non-Hispanic-White	Non-Hispanic-Black	Non-Hispanic-Asian
*N*	2917	526	386	999	805	201
% (SE)	—	16.4 (2.3)	10.7 (1.4)	49.5 (3.3)	18.1 (2.0)	5.3 (0.9)
Age group, %						
20-34 (*n* = 758)	33.7 (1.8)	31.5 (5.2)	23.3 (5.1)	29.6 (3.6)	28.4 (3.9)	32.1 (14.4)
35-49 (*n* = 722)	25.7 (1.1)	44.1 (5.3)	28.7 (5.7)	28.7 (3.6)	30.1 (3.7)	28.0 (8.3)
50-64 (*n* = 744)	23.7 (1.0)	16.2 (3.2)	28.2 (5.7)	30.3 (3.0)	29.7 (3.0)	18.9 (10.4)
65+ (*n* = 633)	17.0 (1.2)	8.2 (2.2)	19.7 (3.6)	11.4 (2.5)	11.9 (2.5)	21.0 (10.9)
Marital status, %						
Married/Living with partner (*n* = 1166)	42.6 (2.1)	55.6 (3.7)	39.4 (9.1)	42.8 (4.2)	27.5 (3.2)	45.3 (7.5)
Separated/Widowed/Divorced (*n* = 1067)	33.5 (1.8)	28.9 (3.6)	35.7 (4.3)	42.9 (3.9)	35.2 (4.0)	33.3 (11.6)
Never married (*n* = 683)	24.0 (2.0)	15.6 (3.2)	24.9 (9.3)	14.3 (3.1)	37.3 (3.7)	21.4 (6.5)
Education, %						
At least some college (*n* = 249)	13.3 (1.3)	1.4 (0.9)	6.6 (4.0)	10.8 (2.6)	8.2 (2.2)	20.5 (10.9)
High School/General Education Development Test (*n* = 865)	43.7 (2.1)	30.6 (4.4)	20.7 (5.9)	54.7 (3.7)	53.2 (4.0)	22.3 (9.6)
Less than High School (*n* = 1034)	43.0 (2.2)	54.9 (4.0)	47.2 (5.6)	22.6 (2.7)	25.0 (2.2)	27.1 (6.0)
Federal assistance (Supplemental Nutrition Assistance Program/ women, infants, and children), % (*n* = 1924)	86.6 (1.6)	89.3 (2.7)	94.1 (2.8)	86.1 (2.5)	87.3 (3.5)	97.3 (1.9)
Household size (n = 2917)	3.3 (0.1)	4.5 (0.2)	3.8 (0.3)	3.3 (0.2)	3.6 (0.2)	4.9 (0.8)
Healthy Eating Index 2015 total score (n = 2917)	49.3 (0.4)	50.6 (0.9)	55.5 (1.4)	47.8 (0.9)	47.2 (0.8)	54.0 (3.5)
Energy intake, kcal (n = 2917)	1748 (18)	1736 (80)	1598 (96)	1787 (51)	1828 (116)	1398 (91)
BMI, kg/m^2^ (n = 2879)	30.5 (0.2)	32.5 (0.8)	30.6 (0.8)	31.9 (0.6)	33.2 (0.8)	24.8 (1.1)
Cardiovascular disease risk factors, %						
High blood pressure or meds (*n* = 2844)	33.5 (1.5)	18.4 (4.1)	33.2 (4.9)	32.9 (4.1)	45.9 (4.0)	13.9 (9.1)
Diabetes or medication (*n* = 2917)	11.2 (0.9)	12.4 (2.4)	19.6 (5.2)	13.2 (2.6)	13.1 (3.3)	12.7 (7.5)
Obesity, BMI ≥30 (*n* = 2879)	47.3 (1.7)	52.2 (6.4)	52.3 (5.3)	53.3 (4.2)	61.7 (3.9)	6.3 (3.1)
High cholesterol (*n* = 1855)	53.5 (1.6)	49.4 (4.4)	58.0 (6.9)	53.1 (4.2)	48.5 (4.0)	49.8 (14.8)
Current smoker (*n* = 2917)	26.2 (1.7)	10.3 (2.9)	11.0 (3.9)	42.0 (4.9)	33.6 (4.5)	5.1 (5.1)

Percentages account for survey sampling weights with SE to account for sampling variability. Counts provided are unweighted*.*

Local patterns were defined by 5 racial and ethnic subgroups. The demographic characteristics of these racial and ethnic subgroups are also described in Table 1. NH-Black females were more likely to be never married. NH-White females were more likely to be separated, widowed, or divorced. All other racial and ethnic subgroups were more likely to be married or living with a partner. Mexican, Other Hispanic, and NH-Asian American females were more likely to have less than a high school education, whereas NH-White and NH-Black females were more likely to have a high school education.

### Global dietary profiles

**FIGURE 2:**
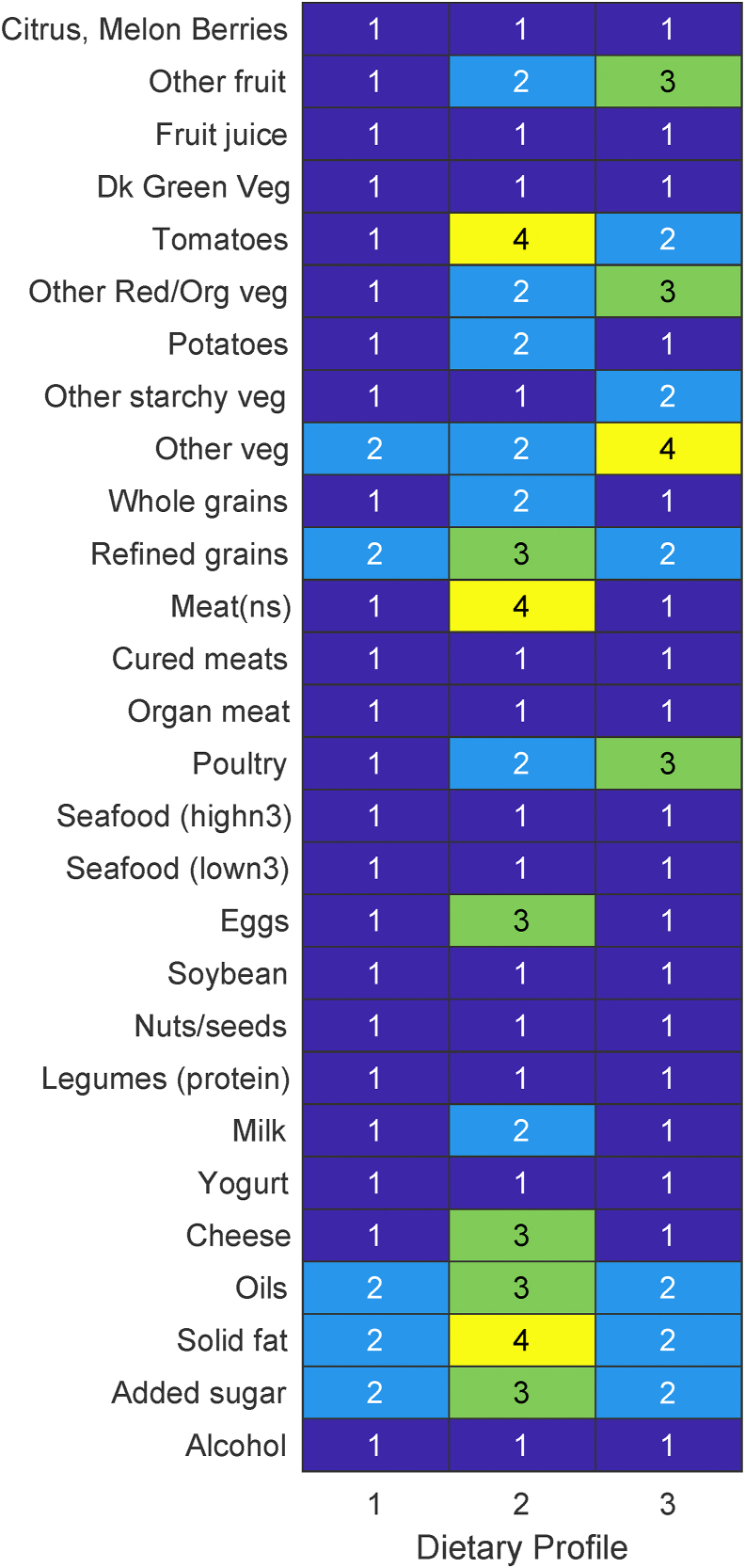
Consumption modal pattern for 3 robust profile clustering-derived global dietary profiles. The level of consumption with the highest probability is highlighted as follows: 1, dark blue (no consumption); 2, light blue (low-level consumption, first tertile: 0%-33%); 3, green (medium-level consumption, second tertile: 34%-66%); 4, yellow (high-level consumption, third tertile: 67%-100%). Food item “meat (ns)” refers to nonspecified meat (beef, veal, pork, lamb, and game).

Three global patterns were derived. Figure 2 illustrates the consumption level with the highest posterior probability for a given food item, given the assignment to a respective profile. We refer to these levels as “consumption modes.” Participants assigned to each of these profiles had a higher probability of not consuming the 12 food items (citrus/melon/berries, fruit juice, dark green vegetables, cured meats, organ meat, high-n3 seafood, low-n3 seafood, soybean products, nuts and seeds, legumes, yogurt, and alcoholic drinks) with probabilities ranging from 26% to 39%. Global profile 1 had the least diet diversity, favoring the consumption of only 5 foods (other vegetables, solid fats, oils, added sugars, and refined grains). The modes of these 5 foods were shared with global profile 3, favoring a low level of consumption. Global profile 2 had the most diet diversity favoring consumption of 15 foods. These foods included a preferred high level of consumption of tomatoes, nonspecified meats, and solid fats and a preferred medium level of consumption of refined grains, eggs, cheese, oils, and added sugar. Global profiles 3 had a medium level of consumption of poultry, other red/orange vegetables, other fruit, and a high level of consumption for other vegetables. The patterns derived from these profiles were weakly defined with the consumption modes ranging from 25% (other red/orange vegetables) to 39% (organ meat). This lower probability of pattern identifiability led to lower precision of assigning participants to a given profile, with a preferred profile being assigned with a probability between 22% and 51%.

### Racial and ethnic-specific local patterns

**FIGURE 3:**
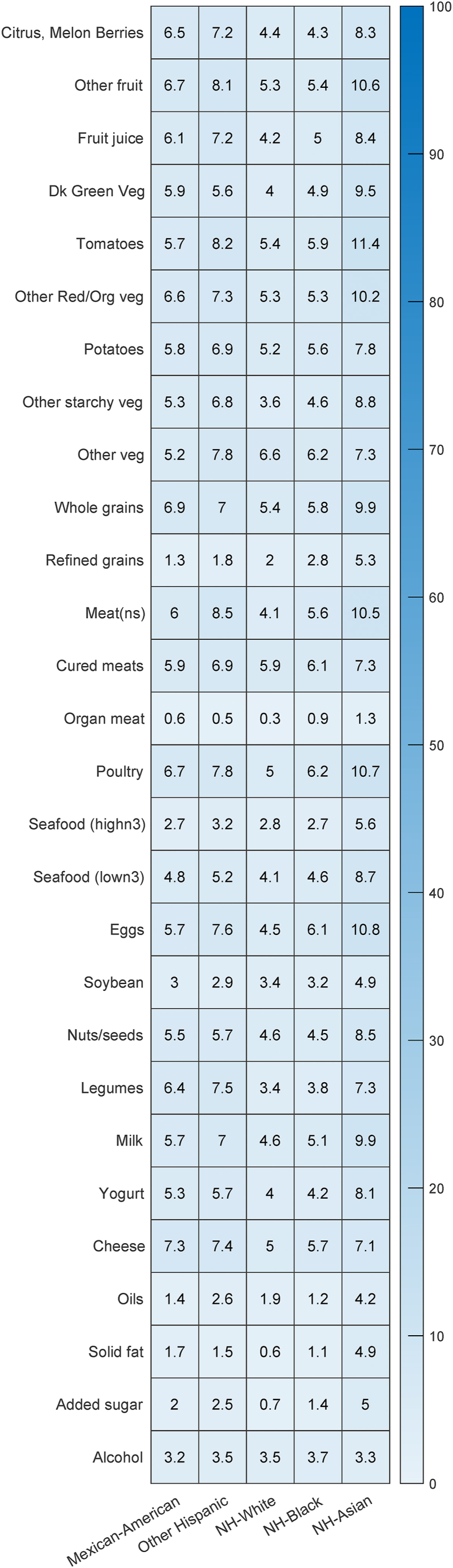
Heatmap illustrating probability of a given food item assuming a pattern at the global level. Values provided are percentages. Foods likely to assume a global pattern will appear darker in blue hue and larger in probability (>70%). Foods likely to assume a localized pattern by the racial and ethnic subgroup will appear lighter in blue hue and smaller in probability (<30%). All foods in this analysis strongly favored a localized pattern. Food item “meat (ns)” refers to nonspecified meat (beef, veal, pork, lamb, and game).

**FIGURE 4:**
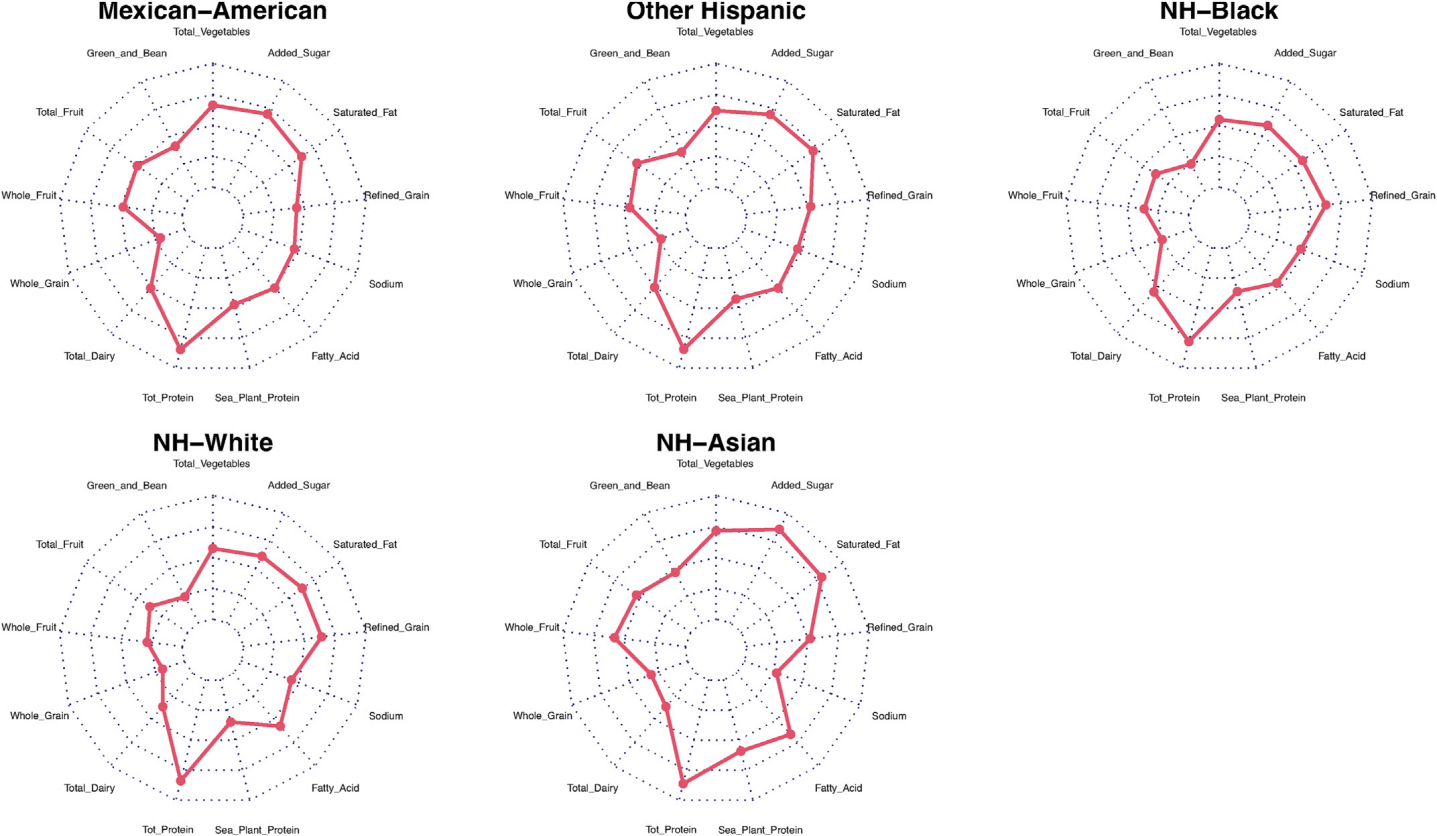
Pattern distributions for robust profile clustering-derived local profiles for each racial and ethnic subgroup. Colored bars denote probability of consumption at that level for a given food: gray, no consumption; yellow, low-level consumption (first tertile); orange, medium-level consumption (second tertile); and red, high-level consumption (third tertile). Racial and ethnic subgroup notations are as follows: M = Mexican–American, H = other Hispanic, W = non-Hispanic-White, B = non-Hispanic-Black, and A = non-Hispanic-Asian American. Food item “meat (ns)” refers to nonspecified meat (beef, veal, pork, lamb, and game).

Under the RPC model, participants assigned to the global patterns were not beholden to all the consumption patterns described at the global level. To determine which food items assumed which pattern, a probability heatmap of a food item (y-axis) assuming the global pattern for each race and ethnicity subgroup (x-axis) was referenced (Figure 3). All 28 food items had less than 12% probability of assuming one of the patterns derived from the global profiles. Given the strong evidence that the consumption patterns of all food items favored patterns at the local level, the locally stratified patterns for each of these racial and ethnic subgroups were examined. Each of the racial and ethnic subgroups had a single local profile to explain the different probabilities of consumption (Figure 4). The probability of consumption at a given level was identified through the vertical bar of relative height for each food item.

Added sugars, solid fats, refined grains, and oils were consumed by all the racial and ethnic subgroups. However, NH-Asian American females were the only subgroup with some participants who did not consume these foods at all during the 2 recall days of record. NH-Black and NH-White females favored a higher consumption of added sugars. Mexican–American females favored higher levels of refined grains consumption. Mexican–American and NH-Asian American females were more likely to favor a higher consumption of other fruit and vegetables. Other Hispanic females showed similar patterns to the Mexican–American females but favored the lower levels of consumption of tomatoes, other vegetables, refined grains, cheese, eggs, and solid fats. NH-Black and NH-White females showed the most similarities in their patterns, but NH-Black females differed with a higher consumption of poultry and fruit juice and NH-White females for the consumption of nuts/seeds, whole grains, milk, and cheese. NH-Asian American females had the most unique dietary pattern compared with the other 4, with a higher consumption of other fruit, dark green vegetables, and other vegetables. Meats and legumes appear to have a mode of nonconsumption for most groups, but NH-Asian American females were least likely to consume cured meats across subgroups and most likely to consume seafood. NH-Black females shared similar higher consumption patterns with NH-Asian American females of low-n3 seafood. Mexican–American females were the only subgroup with greater than 50% probability of consuming legumes. Other Hispanic females had the second highest probability of consuming legumes (44%).

**FIGURE 5:**
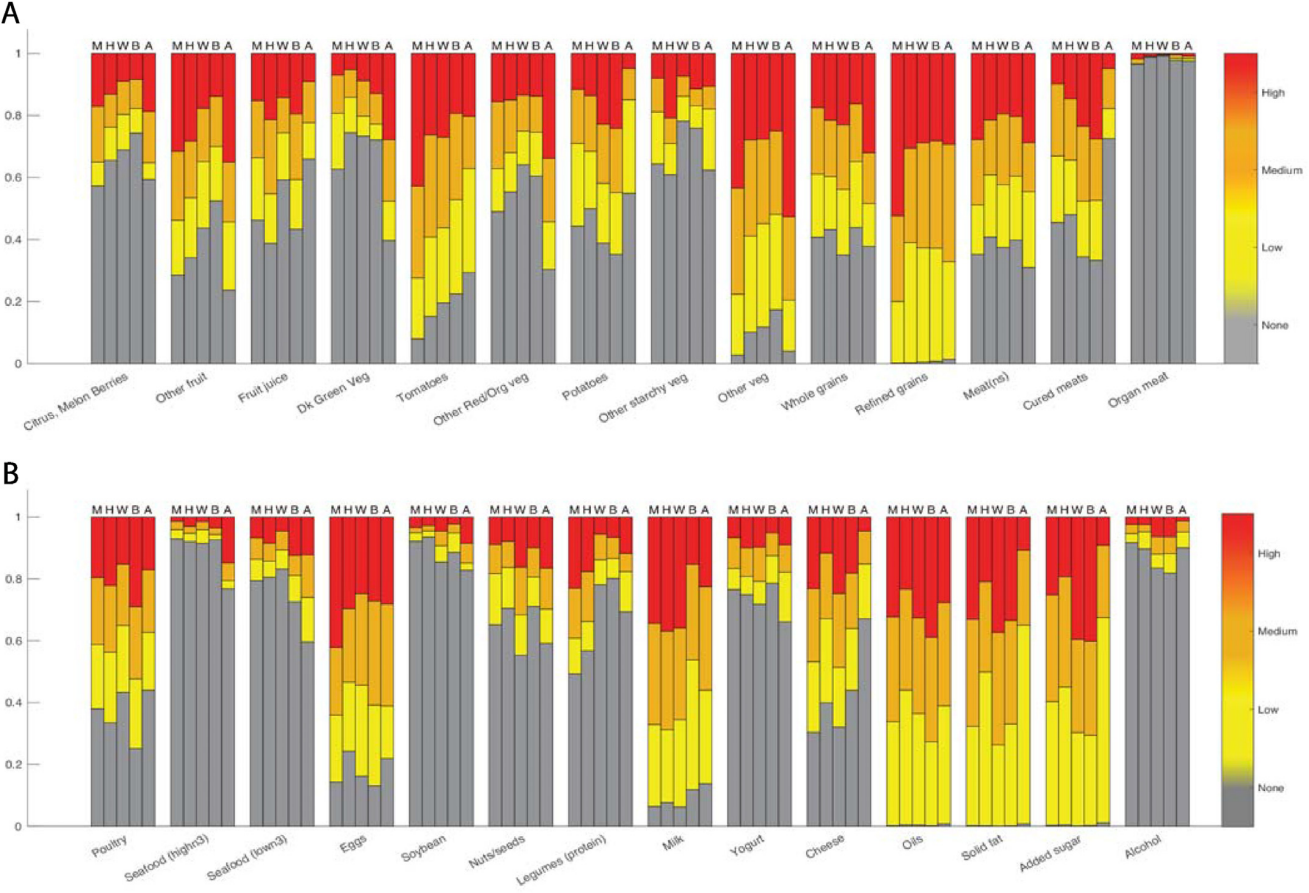
Radar chart of adherence to Healthy Eating Index (HEI)-2015 scores by racial and ethnic subgroup. Shaded gray region illustrates overall mean adherence scores for each of the HEI components. Red boundary line denotes mean component score for each racial and ethnic subgroup. Plots closer to the outer dashed line border indicate greater adherence for that component. Plots shrunk closer to the center indicate lower adherence to that respective component.

We illustrated further on how diet quality differed among the different racial and ethnic subgroups by decomposing the 13 components of the HEI-2015 score (Figure 5). The overall component-specific scores were described with a gray-shaded plot for all low-income female adults. The local dietary patterns derived were consistent with adherence to the HEI components. NH-White and NH-Black females, who comprised 67% of the low-income adult female population, had plots more similar to the overall average. Those scores reflected a lower score adherence to greens/beans and whole grains and a higher score adherence to the total protein levels. NH-Asian American females had the most differentiable plot from the overall average, with higher adherence scores for fruit, vegetables, seafood/plant protein, added sugar, and fat but lower scores for sodium. Mexican–American females also had slightly higher adherence scores to total fruit, whole fruit, and greens and beans components.

## Discussion

The application of this model allowed a deeper look into the consumption behaviors of female adults in the United States who live at or below the 130% poverty income level and into how those behaviors may differ by race and ethnicity. This analysis highlights the following methodological strengths of the RPC model: *1*) the ability to reduce the number of models to better understand the heterogeneity, *2*) to generate a joint-stratified latent class structure with a global LCM identifying patterns shared across multiple subgroups and a stratified LCM to capture the local patterns for each subgroup, *3*) to implement an overfitted latent class model to determine the appropriate number of diet patterns, globally and locally in one single model.

The standard LCM relies on a single model where consumption habits may differ among a general population. Consequently, a demographic that makes up most of the general population (e.g., NH-White female adults) may overpower the consumption behavior patterns reflected masking smaller-sized demographics with differing behaviors. RPC allows greater flexibility by allowing foods and individuals to cluster based on similarities shared among the overall population (global) and similarities based on their subgroup identity (e.g., race and ethnicity). The multilevel clustering is achieved by borrowing information across and within subgroups to better discriminate subgroup-specific differences. Compared with the standard LCM, the RPC improves identifiability, fit to the observed data, and precision of pattern estimation from the model, compared with the standard LCM [15,19].

With expansion of NHANES data collection, starting in 2011, our analysis was able to leverage additional racial and ethnic details (e.g., NH-Asian American, Mexican–American vs Other Hispanic) not previously available in the previous NHANES diet research. This allowed a more in-depth analysis of dietary patterns shared across all low-income female adults and across multiple racial and ethnic subgroups.

Food choices made by low-income female adults are often influenced by cost [32] but are still driven by cultural influences. Legumes and cured meats were 2 of the most differentiating food features reflected in the racial and ethnic subgroups. Consumption of legumes (beans and peas) has been associated with a myriad of nutritional benefits on lowering CVD risk [[33], [34], [35], [36], [37], [38], [39]]. Mexican–American and Other Hispanic females, where beans are a cultural staple of the home, were likely to have higher levels of legumes consumption. Comparatively, NH-Black and NH-White females were less likely to consume legumes and NH-Asian females consumed them, but at lower levels. Cured meat consumption has been associated with a higher risk of CVD [40,41]. NH-Asian female adults had the most uniquely characterized patterns that differed from all other groups. Mexican–American and Other Hispanic females shared similarities in consumption patterns across most foods, with a higher consumption by Mexican–American participants of refined grains, tomatoes, other fruit, and vegetables. Despite poorer expected outcomes and risk factors expected among low-income adults, NH-Asian females had the lowest proportion of high blood pressure, obesity, and smoking status. The diets reflected in this subgroup had a greater proportion of high consumers of fruits, vegetables, and whole grains. NH-White and NH-Black females also shared several similarities in consumption patterns, with a higher consumption of poultry, low n-3 seafood, and cured meats by NH-Black females and a lower consumption of dairy (milk and cheese) and tomatoes. These 2 subgroups shared higher proportions of obesity and smoking status. The reasons behind these dietary differences and relationship with CVD risk remain complex and open for further research.

Previous studies that examined the diets of low-income populations have focused analysis on adherence scores to examine the diet quality, emphasizing the consumption of fruits, vegetables, and whole grains [42,43]. However, Lin et al. focused on the overall low-income population comparing those who did or did not participate in federal assistance programs [42]. Racial and ethnic differences were not considered. Therefore, the mean estimates provided in that analysis are dominated by larger-sized racial and ethnic demographics and may not capture the variation present in smaller-sized subgroups, such as NH-Asian Americans. Leung et al. used earlier NHANES survey cycles that did not have as many racial and ethnic subgroups available compared with 2011. Furthermore, these studies focused analysis on specific foods or nutrients, calculating adjusted mean intake using regression analysis. This isolation of individual foods/nutrients may not reflect how those items are consumed in practice. Our RPC analysis utilizes all foods summarized by the dietary intake tool to examine dietary consumption behaviors using a more flexible latent class approach that can identify localized patterns that deviate from the overall population. For example, findings of previous studies indicated an overall lower intake of fruits and vegetables, whereas our joint-stratified model was able to identify certain racial and ethnic groups where higher or lower intake levels of these items occurred.

As with most models, the output is only as good as the input. Dietary patterns reflected in this model are based on the consumption amounts reported on 2 24-hour dietary recalls by the NHANES participants. The foods reported were summarized using publicly available tools provided by USDA and NHANES [[24], [25], [26], [27]]. These components provide greater insights into food heterogeneity compared with the 13 components used to score the HEI-2015. However, another layer of granularity would help parse out some of the pattern similarities identified across subgroups for certain food components. Our analysis was limited to data sources and tools that were publicly available from NHANES and USDA. Researchers with access to original dietary assessment study data are encouraged to use the RPC model to examine these patterns at a more disaggregated level.

Previous application of RPC has been used on the food propensity questionnaire (FPQ) data, highlighting diet heterogeneity among Hispanic/Latino ethnic background and US geography [19]. Access and use of the FPQ allowed for patterns to be described at a more granular level (e.g., 132 foods) but was limited in only describing the frequency of consumption. Our use of 24-hour recalls provided insights into the amount of food consumption allowing us to characterize the patterns based on relative levels of consumption but limited our knowledge of whether the foods reported were episodically or frequently consumed.

Another limitation in the nutrition studies is measurement of errors, and our model did not account for this. As a result, the cases of underreporting and overreporting of consumption are possible, and patterns should be considered with potential misclassification in mind. Previous validation studies on dietary assessments have concluded that the use of multiple 24-hour recalls are still useful in providing reasonably valid estimates of reported intake, although errors can be substantial for foods consumed episodically [44]. Our analysis focused on relative reported consumption of foods, as opposed to defining diets on an accurate reporting of the amount of food consumption. As a result, the associations we observed between race and ethnicity and dietary patterns are likely to exist but may be understated.

In conclusion, this approach effectively identified heterogeneity in diet quality and foods consumed among subgroups of low-income female adults, defined by race and ethnicity, that have previously not been fully appreciated in overall population diet analysis. With a more recent focus on community precision nutrition, approaches such as the RPC may be of value for targeting efforts to improve the diets of population subgroups at risk of adverse health outcomes.

## Author disclosures

The authors report no conflicts of interest.

## Data Availability

All data and supporting code to reproduce analysis reported are publicly available on GitHub repository: https://github.com/bjks10/RPC/tree/master/NHANES. Source data can be publicly accessed from CDC/NHANES (https://wwwn.cdc.gov/nchs/nhanes/default.aspx) and USDA/ARS (https://www.ars.usda.gov/northeast-area/beltsville-md-bhnrc/betlsville-human-nutrition-research-center/food-surveys-research-group/docs/fped-databases/).

## Appendix A Supplementary data

Multimedia components 1 Multimedia components 1
