# Supplementary material for: Racial and ethnic heterogeneity in diets of low-income adult females in the United States: results from National Health and Nutrition Examination Surveys from 2011 to 2018
Source: Am J Clin Nutr. 2023 Mar 3;117(3):625–34. doi: 10.1016/j.ajcnut.2023.01.008 (PMC10315405; doi:10.1016/j.ajcnut.2023.01.008)
Supplement: Multimedia components 1 [file mmc1.docx]

**SUPPLEMENTARY MATERIALS**

Table 1. List of USDA 37 food pattern components generated from 24-hour recalls collected from NHANES 2011-2018.

| **USDA Food Pattern Component** | **Included** | **Excluded** |
| --- | --- | --- |
| Total Fruit |  | X |
| Citrus, Melon, and Berries | X |  |
| Other Fruits | X |  |
| Fruit juice | X |  |
| Total Vegetables |  | X |
| Dark Green vegetables | X |  |
| Total red and orange vegetables |  | X |
| Tomatoes | X |  |
| Other red and orange vegetables (excludes tomatoes) | X |  |
| Other vegetables | X |  |
| Legumes (beans and peas computed as vegetables) |  | X |
| Total Grains |  | X |
| Whole Grains | X |  |
| Refined Grains | X |  |
| Total protein foods |  | X |
| Total meat, poultry, and seafood |  | X |
| Meat (beef, veal, pork, lamb, game) | X |  |
| Cured meat (frankfurters, sausage, corned beef, cured ham, and luncheon meat from beef, pork, poultry) | X |  |
| Organ meat (from beef, veal, pork, lamb, game, poultry) | X |  |
| Poultry (chicken, turkey, other fowl) | X |  |
| Seafood high in n-3 fatty acids | X |  |
| Seafood low in n-3 fatty acids | X |  |
| Eggs | X |  |
| Soybean products (excludes calcium fortified soy milk and mature soybeans) | X |  |
| Nuts and seeds | X |  |
| Legumes (beans and peas computed as protein) | X |  |
| Total dairy (milk, yogurt, cheese, whey) |  | X |
| Milk (includes calcium fortified soy milk) | X |  |
| Yogurt | X |  |
| Cheese | X |  |
| Oils | X |  |
| Solid fats | X |  |
| Added sugars | X |  |
| Alcoholic drinks | X |  |
